# Physical Restraints in a Mental Hospital Emergency Unit: Facts vs Perceptions of Healthcare Workers

**DOI:** 10.1192/j.eurpsy.2023.400

**Published:** 2023-07-19

**Authors:** E. Sönmez Güngör, E. Poyraz, B. N. Güvendi, M. Eriş, O. Durmaz

**Affiliations:** Psychiatry, Erenköy Mental Health and Neurological Diseases Training and Research Hospital, İstanbul, Türkiye

## Abstract

**Introduction:**

Coercion is a general term used to refer to a series of actions, ranging from involuntary treatments to forced interventions, which can be ethically, legally and clinically challenging for both professionals and service-users. Perception of healthcare professionals on restraint practices is in important factor determining the clinical outcomes.

**Objectives:**

The aim of this study was to determine i) the differences between the estimates of healthcare professionals working in the Emergency Unit of Erenköy Psychiatric and Neurological Training and Research Hospital (Erenköy RSHEAH) regarding physical restraint practices and the real use and outcome values ii) the knowledge, attitudes and opinions of healthcare professionals on such procedures.

**Methods:**

The study was designed as a descriptive cross-sectional online survey. All healthcare professionals working in the Emergency Unit of Erenköy RSHEAH (with a catchment area of 5 million people) who agreed to participate in the study and who were not part of the research team were included. Sociodemographic information, information about working experience, and estimates of physical restraint practices in the last month were questioned. Attitudes and opinions towards these practices were evaluated with 5-point Likert-type questions. Ethical approval was obtained from Erenköy RSHEAH Clinical Ethics Committee (Decision No: 40, 18.07.2022).

**Results:**

A total of 55 healthcare workers (31 trainees, 10 specialist psychiatrists, 6 nurses, 8 security personnel) participated in the study. The mean age of the participants was 32±6.4 years (24-50, min-max) and 52.7% were female. The mean duration of employment in the health sector and current institution were 6.6±5.7 (1-22, min-max), and 4.1±4.1 (1-17, min-max) years, respectively. The estimated mean rate of physical restriction was 13.5±13% (2-60, min-max). However, the actual median rate was 4% (0 - 8.8% min-max) in the same month. The estimated mean duration of physical restraint was reported as 87.8±54.1 (20-300, min-max) minutes, whereas the median actual duration of physical restraint was 60 minutes. No significant relationship was found between the estimates of duration, rates and complication rates of of physical restraint and the duration of professional experience (p>0.05). A significant difference was found between professional groups in terms of restraint duration and complication rate (p<0.05), as well as their opinions regarding the appropriateness of restraint practices with the legal framework (Table 1).

**Image:**

**Figure:**
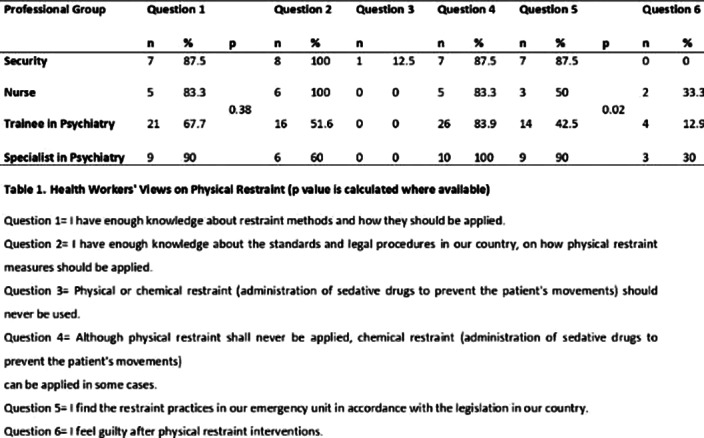

**Conclusions:**

It was observed that healthcare workers had a misperception regarding the rate and duration of physical restraints, which were perceived as higher than the actual values. Thus, the restraint interventions were perceived to be more negative than they actually are. Correction of such misperceptions should become part of the continous educational processes of all professional groups.

**Disclosure of Interest:**

None Declared

